# Distinct Roles of Vav Family Members in Adaptive and Innate Immune Models of Arthritis

**DOI:** 10.3390/biomedicines9060695

**Published:** 2021-06-19

**Authors:** Javier Conde, Isabel Fernández-Pisonero, Myriam Cuadrado, Antonio Abad, Javier Robles-Valero, Xosé R. Bustelo

**Affiliations:** 1 Mechanisms of Cancer Program, Centro de Investigación del Cáncer, CSIC-University of Salamanca, 37007 Salamanca, Spain; javier.conde.aranda@sergas.es (J.C.); mfernandezpisonero@usal.es (I.F.-P.); mcuadrado@usal.es (M.C.); aabad@usal.es (A.A.); jrobles@usal.es (J.R.-V.); 2 Instituto de Biología Molecular y Celular del Cáncer, CSIC-University of Salamanca, 37007 Salamanca, Spain; 3 Centro de Investigación Biomédica en Red de Cáncer (CIBERONC), CSIC-University of Salamanca, 37007 Salamanca, Spain

**Keywords:** effector T cells, helper T cells, cytotoxic T cells, neutrophils, joint, mouse model

## Abstract

Genetic evidence suggests that three members of the VAV family (VAV1, VAV2 and VAV3) of signal transduction proteins could play important roles in rheumatoid arthritis. However, it is not known currently whether the inhibition of these proteins protects against this disease and, if so, the number of family members that must be eliminated to get a therapeutic impact. To address this issue, we have used a collection of single and compound Vav family knockout mice in experimental models for antigen-dependent (methylated bovine serum albumin injections) and neutrophil-dependent (Zymosan A injections) rheumatoid arthritis in mice. We show here that the specific elimination of Vav1 is sufficient to block the development of antigen-induced arthritis. This protection is likely associated with the roles of this Vav family member in the development and selection of immature T cells within the thymus as well as in the subsequent proliferation and differentiation of effector T cells. By contrast, we have found that depletion of Vav2 reduces the number of neutrophils present in the joints of Zymosan A-treated mice. Despite this, the elimination of Vav2 does not protect against the joint degeneration triggered by this experimental model. These findings indicate that Vav1 is the most important pharmacological target within this family, although its main role is limited to the protection against antigen-induced rheumatoid arthritis. They also indicate that the three Vav family proteins do not play redundant roles in these pathobiological processes.

## 1. Introduction

The Vav family is a group of tyrosine-phosphorylated signaling molecules that play critical regulatory roles downstream of protein tyrosine kinases. In mammals, this family is composed of three members, Vav1, Vav2 and Vav3 [1,2,3]. Vav1, the founding member of the family, shows under physiological conditions a hematopoietic-specific expression [4,5]. By contrast, the other two members have more ubiquitous expression patterns [6,7]. The main function of these proteins is to act as guanosine nucleotide exchange factors for Rho GTPases, a catalytic function that facilitates the transition of the downstream GTPases from the inactive (GDP-bound) to the active (GTP-bound) state [7,8,9]. They also play noncatalytic, adaptor-like functions that are mediated by interactions with a large variety of downstream protein partners [2]. These adaptor functions are cell type- and, in most cases, Vav family-member-specific [1,2,3]. These proteins play important signaling roles in immune cells implicated in rheumatoid arthritis such as T cells, B cells, macrophages and neutrophils [2]. In line with this, a number of observations suggest that some Vav family members can also be connected to this disease. For example, recent studies have linked *VAV1* single nucleotide polymorphisms (SNP) with the degree of severity of rheumatoid arthritis, multiple sclerosis and other autoimmune diseases [10,11,12]. SNPs found in the rat *Vav1* locus have also been associated with the severity of autoimmune encephalomyelitis (the rat model for multiple sclerosis) and pristane-induced autoimmune arthritis [11,12]. Lastly, using triple *Vav1*^–/–^;*Vav*2^–/–^;*Vav3*^–/–^ knockout mice, Faccio and coworkers have shown that the elimination of the three Vav family members can block the development of a neutrophil-dependent model of rheumatoid arthritis in mice [13]. It is not currently known, however, whether this function requires the involvement of the entire Vav family or specific family members. It is not known either whether those functions can be generalized to all types of innate system-associated rheumatoid arthritis.

The foregoing data suggest that the inhibition of Vav family proteins could be a potential therapeutic avenue in rheumatoid arthritis and related diseases. However, up to now, very little information is available regarding the specific roles of Vav family proteins in this pathobiological process. We do not know either whether the inactivation of these proteins can protect against rheumatoid arthritis development and, in that were the case, if we need to inhibit one or more than one family member to obtain such protective effects. Finally, we still do not know whether these proteins play roles in the different types of experimental arthritic models. In this work, we have used our collection of single and compound Vav family knockout mice to tackle those issues in two experimental models for rheumatoid arthritis that are heavily dependent on the engagement of cells from either the adaptive or the innate immune system. Our findings indicate that, among the three family proteins, Vav1 is probably the best therapeutic candidate for the treatment of T cell-dependent rheumatoid arthritis.

## 2. Materials and Methods

### 2.1. Mice

Single *Vav1*^–/–^, single *Vav2*^–/–^, single *Vav3*^–/–^, double *Vav2*^–/–^; *Vav3*^–/–^ and triple *Vav1*^–/–^; *Vav2*^–/–^; *Vav3*^–/–^ knockout mice (C57BL/10 genetic background) have been previously described [14,15,16,17]. All animals used in the experiments were 10 to 12 weeks old at the beginning of the experiments. All mouse experiments were performed according to protocols approved by the Bioethics Committee of the University of Salamanca (18 December 2018, approval code #315) as well as the animal experimentation authorities of the autonomous government of Castilla y León (11 April 2019, approval code #315) (Spain). We have not utilized patients or patient-derived samples in this work.

### 2.2. Antigen-Induced Arthritis

Animals of the indicated genotypes were immunized intradermally with 100 μg of methylated bovine serum albumin (mBSA) (Sigma-Aldrich, Saint Louis, MO, USA; Cat. No. A1009). To this end, mBSA was diluted in phosphate-buffered saline solution and emulsified in an equal volume of complete Freund’s adjuvant supplemented with 5 mg/mL heat-killed *Mycobacterium tuberculosis* (Chondrex, Woodinville, WA, USA; Cat. No. 7023). After seven days, animals were injected again with the same solution as above. Arthritis was then induced on day 21 through the intra-articular injection of 100 μg of mBSA in a total volume of 10 μL phosphate-buffered saline solution in the right knee joint of each experimental animal. As a control, the left knee joint of each animal was injected with an equal volume of phosphate-buffered saline solution. The development of arthritis was periodically monitored by measuring the transverse diameter of the joint on the fourth, eighth and twelfth postinjection days using a caliper. Mice were sacrificed 12 days later for subsequent histological and cytological analyses.

### 2.3. Histological Determinations

Joint samples were collected, fixed in 4% paraformaldehyde (PanReac AppliChem-ITW Reagents, Castellar del Vallès, Spain; Cat. No. 252931), decalcified for 72 h with Osteosoft (Merck, Darmstadt, Germany; Cat. No. 101728), embedded in paraffin, and cut in 5 µm thick sections. Sagittal sections of the whole joint were stained with either hematoxylin/eosin or Safranin-O to visualize tissue histology and cartilage damage, respectively. In the latter case, sections were counterstained with Fast Green. Images were captured using an BX51 microscope coupled to an DP70 digital camera (Olympus, Shinjuku-ku, Tokyo, Japan). Pathological severity was scored blindly by an independent pathologist in our Pathology Unit. In the case of antigen-induced arthritis, synovial inflammation and cartilage erosion were both evaluated using a scoring system ranging from 0 to 4, where 0 means normal status, 1 means minimal disease, 2 means moderate disease, 3 means severe disease and 4 means very severe disease. In the case of Zymosan A-induced arthritis, we scored three independent parameters: synovial hyperplasia (from 0 [normal] to 3 [most severe hyperplasia]), inflammatory cell infiltration (from 0 [no infiltration] to 3 [most severe infiltration]) and cartilage damage (from 0 [sections fully stained with Safranin-O] to 3 [total loss of Safranin-O staining]). The average of these three scores was utilized to obtain the final histology score. The percentage of neutrophils infiltrated in the synovium was calculated using the neutrophil/synovial fibroblast ratio obtained in several independent areas of the interrogated synovial tissue.

### 2.4. Cell Isolation

In the case of blood cells, the samples were incubated with 0.17 M NH_4_Cl and washed three times with phosphate-buffered saline solution to remove erythrocytes. In the case of splenic and inguinal lymph node cells, the tissues were mechanically homogenized in 3 mL of phosphate-buffered saline solution supplemented with 2% bovine serum albumin and 0.5 mM EDTA using a Dispomix Drive homogenizer (Medic Tools AG, Solothurn, Switzerland; Cat. No. 900020.00) and gentleMACS C tubes (Myltenyi Biotec, Bergisch Gladbach, Germany; Cat. No. 130-096-334) and then incubated subjected to an erythrocyte lysis step as above. In the case of analyses using cells from the knee joints, we eliminated the skin and, subsequently, incubated the joints for 6 h with collagenase (1 mg/mL).

### 2.5. Flow Cytometry

Isolated cells were stained with FITC-labeled CD4 (BD Pharmingen, Franklin Lakes, NJ, USA; Cat. No. 553729), V500 Horizon-labeled CD4 (BD Pharmingen, Cat. No. 560783), PB-labeled CD8 (BD Pharmingen, Cat. No. 558106), PE-Cy7-labeled CD3 (BD Pharmingen, Cat. No. 560591), PE-labeled CD11b (BD Pharmingen, Cat. No. 553311), APC-labeled F4/80 (eBioscience, San Diego, CA, USA; Cat. No. 50-4801-82), FITC-labeled Ly6G (eBioscience, Cat. No. 11-5931-82), FITC-labeled CD45 (eBioscience, Cat. No. 11-0451-82), PE-labeled CD69 (eBioscience, Cat. No. 12-0691-81), APC-labeled CD86 (BD Pharmingen, Cat. No. 558703), PerCP-Cy5.5-labeled B220 (eBioscience, Cat. No. 45-0452-82), PerCP-Cy5.5-labeled IFNγ (BD Pharmingen, Cat. No. 560660), PE-Cy7 labeled TNFα (eBioscience, Cat. No. 25-7321-82), APC-labeled interleukin 4 (BD Pharmingen, Cat. No. 554436) and PE-labeled interleukin 17 (BD Pharmingen, Cat. No. 561020) antibodies.

### 2.6. Quantitative RT-PCR

Knee joints were homogenized with a mortar in gentleMACS M containing 1 mL of NZYol (NZYtech, Cat. No. MB18501). Total RNA was then isolated using the RNeasy Mini Kit (Qiagen, Hilden, Germany; Cat. No. 74104) and subjected to qRT-PCR analyses using the Power SYBR Green RNA-to-CT™ 1-Step Kit (Applied BioSystems, Waltham, MA, USA; Cat. No. 4389986) and the StepOnePlus Real-Time PCR System (Applied BioSystems, Cat. No. 4376600) according to the supplier’s instructions. Raw qRT-PCR data were analyzed using the StepOne software v2.1 (Applied BioSystems) using the levels of the *Gapdh* mRNA as the internal normalization control. Primers used for these experiments included: 5′-CGT CAG CCG ATT TGC TAT CT-3′ (forward for *Tnfa*), 5′-CGG ACT CCG CAA AGT CTA AG-3′ (reverse for *Tnfa*), 5′-TCA AGT GGC ATA GAT GTG GAA GAA-3′ (forward for *Ifng*), 5′-TGG CTC TGC AGG ATT TTC ATG-3′ (reverse for *Ifng*), 5′-CTC CAG AAG GCC CTC AGA CTA C-3′ (forward for *Il17*), 5′-GGG TCT TCA TTG CGG TGG-3′ (reverse for *Il17*), 5′-CAG TTT GGT AGC ATC CAT CAT TTC T-3′ (forward for *Il6*), 5′-GCA CAG GGT CAT CAT CAA AGA C-3′ (reverse for *Il6*), 5′-ACG GAC CCC AAA AGA TGA AGG GCT-3′ (forward for *Il1b*), 5′-GGG AAC GTC ACA CAC CAG CAG G-3′ (reverse for *Il1b*), 5′-TGC ACC ACC AAC TGC TTA GC-3′ (forward for *Gapdh*) and 5′-TCT TCT GGG TGG CAG TGA TG-3′ (reverse for *Gapdh*).

### 2.7. T and B Cell Activity Assays

Inguinal lymph node- and spleen-derived cells were harvested on day 21 after the first mBSA immunization step and, after generating single cells suspensions as above, seeded in 6-well plates (1.5 × 10^6^ cells/well) containing RPMI 1640 medium (Sigma-Aldrich, Cat. No. R8758) supplemented with penicillin (100 U/mL), streptomycin (100 μg/mL) and 5 × 10^−5^ M β-mercaptoethanol (Sigma-Aldrich, Cat. No. 3148). Cells were maintained in a humidified incubator at 37 °C and a 5% CO_2_ atmosphere.

For T cell proliferation assays, inguinal lymph node cells were incubated with mBSA (50 μg/mL), antibodies to CD3 (2 μg/mL; BD Pharmingen, Cat. No. 553057) or medium alone for 48 h. For B cell proliferation assays, splenic lymphocytes were treated with antibodies to CD40 (5 μg/mL; BD Pharmingen, Cat. No. 553721) or medium alone for 48 h. Two hours before the end of each treatment, cells were incubated with EdU (10 μM) and the proliferative fraction identified using an EdU kit (Invitrogen, Walthan, MA, USA; Cat. No. C10424) and flow cytometry according to the manufacturer’s instructions. Surface staining with antibodies to CD3, CD4, CD8 or B220 was performed according to manufacturer’s recommendations.

For T cell activation assays, inguinal lymph node cells harvested on days 0 or 21 of the first immunization step were restimulated ex vivo using mBSA (50 μg/mL), antibodies to CD3 (2 μg/mL) and to CD28 (2 μg/mL) (BD Pharmingen, Cat. No. 553295) or maintained in medium alone for 24 h. Cells were incubated with Brefeldin A (5 μg/mL; Sigma-Aldrich, Cat. No. 7651) 4 h prior to final harvesting. Cells were incubated with antibodies to CD4 and CD8 as above, fixed, permeabilized using the BD Cytofix/Cytoperm reagent (BD Biosciences, Cat. No. 554722) and incubated for 45 min with antibodies to the indicated proteins. For B cell activation studies, splenocytes harvested on day 21 after the first immunization step were incubated with either medium alone or antibodies to IgM (Jackson ImmunoResearch, West Grove, PA, USA; Cat. No. 115-006-020) for 48 h. Cells were then harvested and stained with surface antibodies to CD69 and CD86 and analyzed by flow cytometry.

### 2.8. Zymosan A-Induced Arthritis

Zymosan A (20 mg/mL) from *Saccharomyces cerevisiae* (Sigma-Aldrich, Cat. No. Z4250) was solubilized in phosphate-buffered saline solution by extensive boiling and sonication. Nine μL of that suspension were then injected into the right knee joint of each experimental animal. As a control, the left joints of the same animals were injected with an equal amount of sterile phosphate-buffered saline solution. The progression of arthritis was then monitored by measuring with a caliper the transverse diameter of the knee joints at the first, third and seventh postinjection days.

### 2.9. Statistical Analyses

Student’s *t*-tests for the comparison of two groups or ANOVA followed the appropriate post-hoc test for the comparison of multiple groups were applied to the data generated in this study by using GraphPad software. Normality tests found mostly normal distribution of outcome parameters within groups. Sample size and number of independent experiments for each experiment are indicated in the appropriate figure legend. Experimental values in graphs are provided as mean ± SEM. Results with *p*-values ≤ 0.05 were considered statistically significant.

## 3. Results

### 3.1. Vav1 Deficiency Decreases the Severity of Antigen-Induced Arthritis

**Figure 1:**
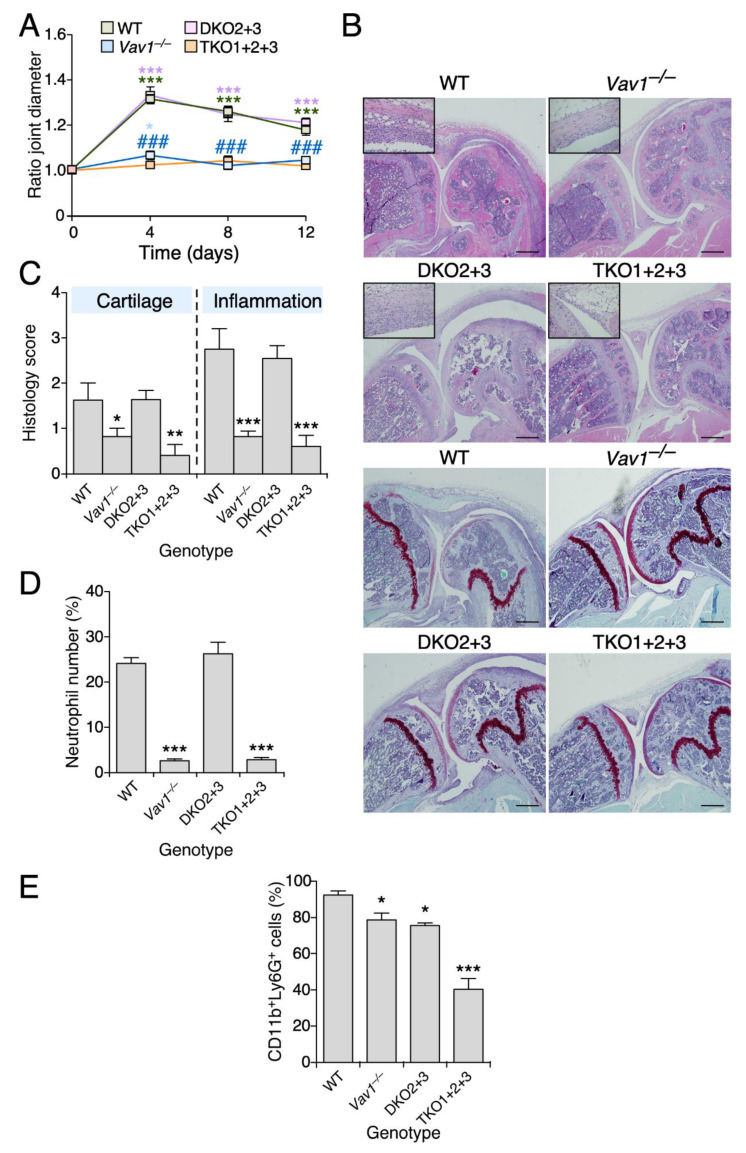
The Vav1 deficiency decreases the severity of antigen-induced arthritis. (**A**) Time-dependent evolution of the ratio joint diameter in animals of the indicated genotypes upon being treated with either mBSA or placebo. WT, wild-type mice; DKO2 + 3, *Vav2*^–/–^; *Vav3*^–/–^ mice; TKO1 + 2+3, *Vav1*^–/–^; *Vav2*^–/–^; *Vav3*^–/–^ mice. This notation has been used in the rest of the panels and figures of this work. (**B**) Representative images of histological sections from arthritic knee joints of mice of indicated genotypes that were stained with hematoxylin-eosin in the absence (four top panels) or presence (four bottom panels) of Safranin-O Fast Green. Scale bar, 500 µm. Insets show a higher magnification of the synovial tissue present in the joint section of each appropriate panel. (**C**) Levels of cartilage damage (**left**) and inflammation status (**right**) 12 days after the intra-articular injection of mBSA in the joints of animals of the indicated genotypes. (**D**) Quantification of the neutrophils infiltrated into the synovium (expressed as the percentage of neutrophils versus synovial fibroblasts) in the joints injected with mBSA of mice of indicated genotypes. (**E**) Percentage of circulating neutrophils in blood evaluated by flow cytometry. Data shown in panels A, C to E represent the mean ± SEM. *, *p* ≤ 0.05; **, *p* ≤ 0.01; ***, *p* ≤ 0.001 relative to either experimental time-point 0 (**A**) or the values obtained with WT mice (**C**–**E**). ^###^, *p* ≤ 0.001 relative to WT (**A**). *n* = 10 (WT), 7 (*Vav1*^–/–^ mice), 11 (*Vav2*^–/–^; *Vav3*^–/–^ mice) and 9 (*Vav1*^–/–^; *Vav2*^–/–^; *Vav3*^–/–^ mice).

To investigate the contribution of the three Vav family proteins to antigen-induced arthritis, we compared the joint swelling induced by the intra-articular injection of methylated bovine serum albumin (mBSA) in single *Vav1*^–/–^, double *Vav2*^–/–^; *Vav3*^–/–^ and triple *Vav1*^–/–^; *Vav2*^–/–^; *Vav3*^–/–^ knockout mice. This experimental method elicits an arthritic state that is heavily dependent on the activation of the adaptive immune system [18]. We could not find any significant alteration in the mBSA-induced joint swelling in double *Vav2*^–/–^; *Vav3*^–/–^ mice when compared to controls (Figure 1A). By contrast, the joints from single *Vav1*^–/–^ and the triple *Vav1*^–/–^; *Vav2*^–/–^; *Vav3*^–/–^ knockout animals are totally protected against such inflammatory effects (Figure 1A). Consistent with this observation, we found that the histology of the mBSA-injected knee joints from both *Vav1*^–/–^ and *Vav1*^–/–^; *Vav2*^–/–^; *Vav3*^–/–^ knockout mice do not show the typical pathological signs of an arthritic response, such as the increase in synovial thickness (Figure 1B), cartilage degradation (Figure 1B,C) and presence of high numbers of infiltrating inflammatory cells (Figure 1B,D). By contrast, all those defects are clearly observed in the sections from the rest of the genotypes interrogated in this study (Figure 1B–D). The inflammatory defects are not associated with the lack of recruitment of neutrophils, since we could only find a strong reduction in the number of circulating neutrophils in the case of mBSA-treated *Vav1*^–/–^; *Vav2*^–/–^; *Vav3*^–/–^ mice (Figure 1E). These results indicate that Vav1 is the only Vav family member that substantially contributes to antigen-induced arthritis development.

### 3.2. Vav1 Deficiency Impairs the Infiltration of CD4^+^ T Cells and Macrophages in mBSA-Treated Joints

**Figure 2:**
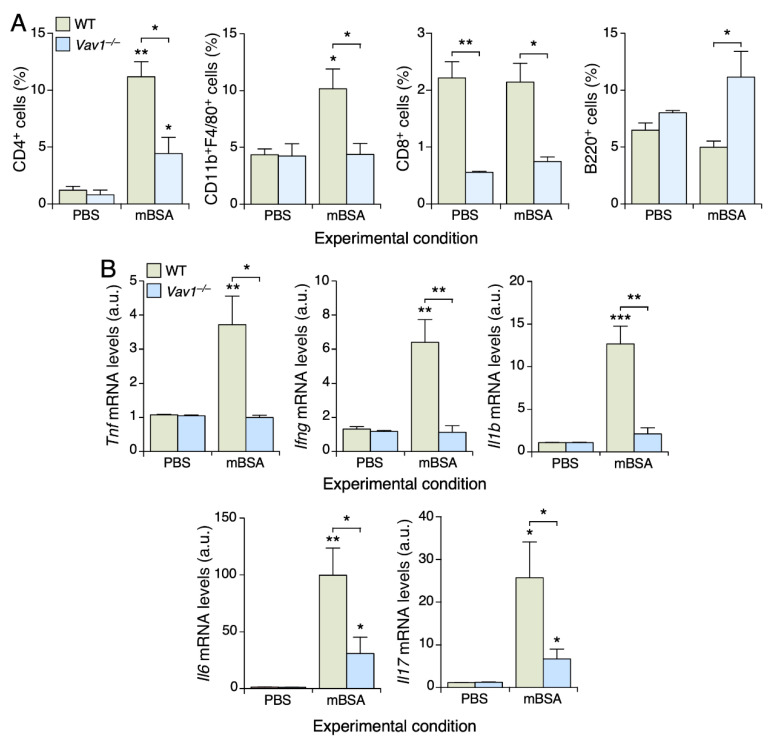
The Vav1 deficiency impairs the infiltration of CD4^+^ T cells and macrophages in mBSA-treated joints. (**A**) Determination by flow cytometry of the infiltration levels of indicated cell types in placebo- and mBSA-injected knee joints of mice of the indicated genotypes. PBS, phosphate-buffered saline solution. (**B**) Levels of indicated transcripts in the knee joints from mice of indicated genotypes (inset) and under the specified experimental conditions (bottom). Values are shown relative to the abundance of each transcript in the placebo-injected WT samples, which were given an arbitrary number (a.u.) of 1. Data shown in panels A and B represent the mean ± SEM. *, *p* ≤ 0.05; **, *p* ≤ 0.01; ***, *p* ≤ 0.001 relative to either placebo-injected joint of animals of the same genotype or relative to the respective WT control (in brackets). *n* = 4 in each experimental condition.

To explore the presence of other hematopoietic cells in the joints of *Vav1*^–/–^ mice, we obtained cell homogenates from animals of the indicated genotypes and characterized them by flow cytometry. In the case of WT mice, we observed increased levels of both CD4^+^ T cells and macrophages in the mBSA-treated joints when compared to the control ones (Figure 2A, two left panels). This is specific, since we did not detect any statistically significant change in the numbers of either CD8^+^ (Figure 2A, third panel from left) or B (Figure 2A, right panel) lymphocytes. In the case of *Vav1*^–/–^ mice, we found that the infiltration of both CD4^+^ T cells and macrophages is reduced and totally abrogated in the mBSA-treated joints, respectively (Figure 2A, two left panels). We also observed that the joints from *Vav1*^–/–^ mice display lower levels of CD8^+^ T cells regardless of whether they are injected with placebo or mBSA (Figure 2A, third panel from left). This is probably due to the partial T lymphopenic state exhibited by these mice [2,17].

In line with the reduced levels of antigen-induced arthritis in *Vav1*^–/–^ mice, we also found using quantitative RT-PCR that the expression of transcripts encoding cytokines involved in the regulation of different immune cell types that participate in the deterioration of the joint tissue is totally eliminated (in the case of *Tnfa*, *Ifng*, *Il1b*) or highly reduced (in the case of *Il6* and *Il17*) in the mBSA-injected joints of *Vav1*^–/–^ mice (Figure 2B).

### 3.3. The Vav1 Deficiency Impairs T Cell Proliferation and Differentiation

**Figure 3:**
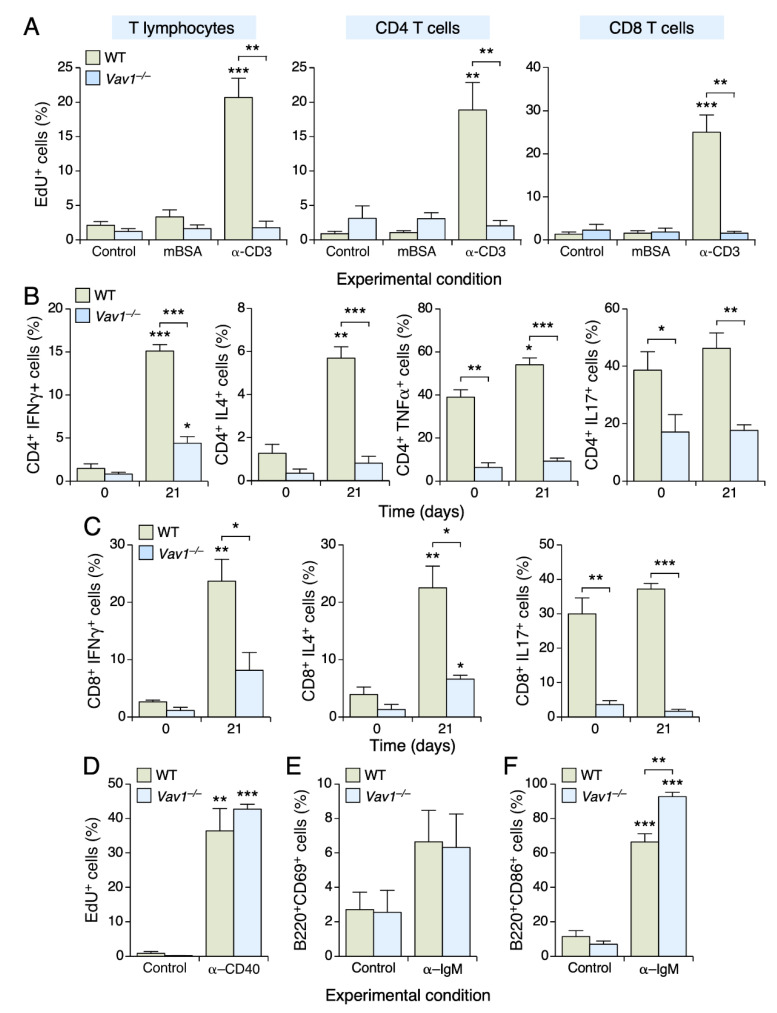
The Vav1 deficiency impairs T cell proliferation and differentiation. (**A**) Percentage of EdU^+^ cells present in the T cell populations (top) from mice of indicated genotypes (inset). The experimental conditions used in each case are indicated at the bottom. (**B**) Percentage of lymph node CD4^+^ T_H1_ (IFNγ^+^ and TNFα^+^), T_H2_ (Il4^+^) and T_H17_ (Il17^+^) cells in mice of the indicated genotypes at the indicated times upon the first intra-dermal immunization with mBSA. Note that cells have been costimulated with antibodies to both CD3 and CD28. (**C**) Percentage of lymph node CD8^+^ T_C1_ (IFNγ^+^), T_C2_ (Il4^+^) and T_C17_ (Il17^+^) generated and stimulated as in B. (**D**) Percentage of EdU^+^ B cells in the indicated experimental animals and conditions. (**E**,**F**) Percentage of B220^+^ B lymphocytes expressing the activation markers CD69 (**E**) and CD86 (**F**) obtained from animals of the indicated genotypes. The experimental conditions used are indicated at the bottom. Data shown in panel A to F represent the mean ± SEM. *, *p* ≤ 0.05; **, *p* ≤ 0.01; ***, *p* ≤ 0.001. Values are given relative to the values obtained in each genotype control (**A**,**D**,**F**), in CD3- (**A**) or IgM-stimulated (**D**–**F**) WT cells, in the experimental time point 0 of animals belonging to the same genotype (**B**,**C**) or in the appropriate time point using WT cells (**B**,**C**). *n* = 3 independent experiments in each case.

Given the role of effector helper (T_H_) and cytotoxic (T_C_) T cells in the development of antigen-induced arthritis [18,19,20], we next investigated the role of Vav1 in the proliferation and differentiation of those T cell lineages. Since the number of cells from joint homogenates was too small to carry out these experiments, we resorted to the use of inguinal lymph node cells in these experiments. To this end, cells were harvested at day 21 upon the first immunization step with mBSA and then maintained in cell culture in the presence of either mBSA or antibodies to CD3 to stimulate their proliferation. Regardless of the genotype analyzed, we did not find any proliferative response in unfractionated CD4^+^ and CD8^+^ T cells in the presence of mBSA (Figure 3A). The addition of antibodies to CD3 does trigger a robust proliferative response in WT cells. However, as expected [2,21], this effect is not observed in the case of *Vav1*^–/–^ CD4^+^ and CD8^+^ T lymphocytes (Figure 3A).

To assess the role of Vav1 in the differentiation of T cells into effector T_H_ and T_C_ cells, we cultured lymph node-derived T cells in the presence of stimulation agents and, subsequently, characterized the effector T cell lineages generated using flow cytometry techniques. Under those conditions, we found that CD4^+^ T cells preferentially differentiate into IFNγ^+^ T_H1_ (Figure 3B, left panel) and IL4^+^ T_H2_ (Figure 3B, second panel from left) cells. A minor increase in TNFα^+^ T_H1_ cells is also observed (Figure 3B, third panel from left). By contrast, we could not detect any significant polarization towards the T_H17_ lineage (Figure 3B, right panel). All the foregoing differentiation processes are severely impaired in the case of *Vav1*^–/–^ CD4^+^ T cells (Figure 3B, three left panels). The Vav1 deficiency also reduces the basal numbers of TNFα^+^ T_H1_ cells (Figure 3B, second panel from left) and T_H17_ (Figure 3B, right panel) cells present under nonstimulated conditions, further underscoring the important role of Vav1 in the differentiation of T_H_ cell lineages. In the case of effector T_C_ cells, we observed that our culture conditions promote the differentiation of both IFNγ^+^ (Figure 3C, left panel) and IL4^+^ (Figure 3C, middle panel) but not IL17^+^ (Figure 3C, right panel) T_C_ cells. By contrast, the differentiation of both the IFNγ^+^ and IL4^+^ T_C_ cells is severely impaired in the absence of Vav1 (Figure 3C, left and middle panels). Interestingly, we also observed that the basal numbers of the T_C17_ cell lineage are highly reduced in the lymph nodes from *Vav1*^–/–^ mice (Figure 3C, right panel). Although B cells play critical roles in arthritis development [22], we could not find any proliferative (Figure 3D) or activation (Figure 3E,F) defect in *Vav1*^–/–^ B lymphocytes. These results demonstrate that the lack of Vav1 causes a dramatic impairment in the generation of both T_H_ and T_C_ cell functions.

### 3.4. Vav2, but Not the Rest of Vav Family Proteins, Plays Roles in Zymosan A-Induced Arthritis

**Figure 4:**
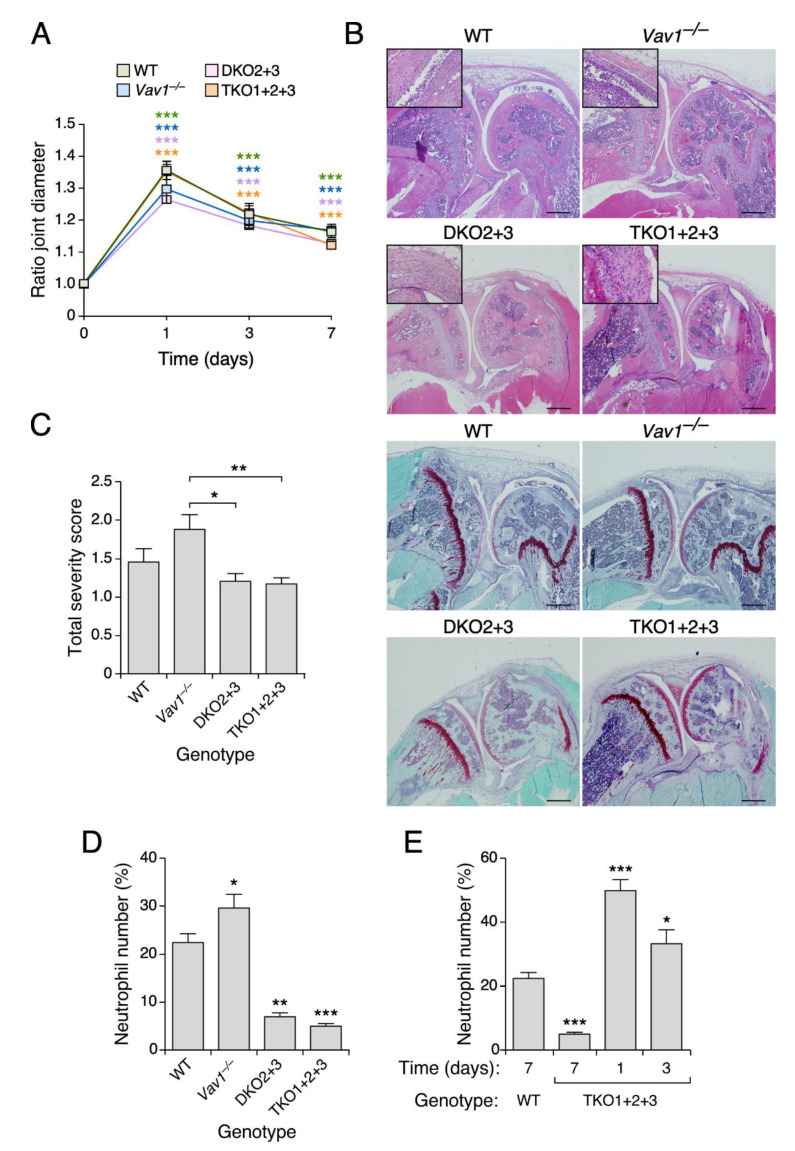
The Vav family is important for sustained inflammatory responses during Zymosan A-induced arthritis. (**A**) Evolution of the ratio joint diameter in animals of the indicated genotypes treated with Zymosan A. (**B**) Representative images of histological sections of knee joints from mice of the indicated genotypes that were stained with hematoxylin-eosin (four top panels) and Safranin-O Fast Green (four bottom panels). Scale bar, 500 µm. Insets show a higher magnification of the synovial tissue present in the joint section of each appropriate panel. (**C**) Histological scores for arthritis severity obtained by evaluating hyperplasia, inflammation and cartilage damage 7 days after the intra-articular injection of Zymosan A. (**D**) Quantification of the neutrophils present in the synovium (expressed as the percentage of neutrophils versus synovial fibroblasts). (**E**) Quantification of the neutrophils infiltrated into the synovium, expressed as the percentage of neutrophils versus synovial fibroblasts. Data shown in panels (**A**,**C**–**E**) represent the mean ± SEM. *, *p* ≤ 0.05; **, *p* ≤ 0.01; ***, *p* ≤ 0.001. In (**A**,**C**,**D**), *p*-values are given relative to the data obtained at time 0 (**A**), in *Vav1*^–/–^ mice (**C**) and in WT controls (**D**,**E**). *n* = 13 (WT), 16 (*Vav*1^–/–^ mice), 11 (*Vav2*^–/–^; *Vav3*^–/–^) and 13 (*Vav1*^–/–^; *Vav2*^–/–^; *Vav3*^–/–^) mice. In (**E**), *n* = 6 (WT, day 7), 6 (*Vav1*^–/–^; *Vav2*^–/–^; *Vav3*^–/–^, day 7), 7 (*Vav1*^–/–^; *Vav2*^–/–^; *Vav3*^–/–^ mice, day 1) and 6 (*Vav1*^–/–^; *Vav2*^–/–^; *Vav3*^–/–^, day 3) mice.

To investigate the role of Vav family proteins in joint inflammatory events mediated by the innate immune system, we analyzed our collection of Vav family knockout models using the Zymosan A-induced arthritis model [23]. We did not find any statistically significant change in the swelling of the joints independently of the mouse genotype used (Figure 4A). Consistent with this, we observed using histological analyses that the Zymosan A-injected joints display similar levels of hyperplasia and Safranin-O staining, regardless of the genotype of the mouse involved (Figure 4B). In fact, we found a slightly worse severity score in the case of *Vav1*^–/–^ mice than in the rest of the genotypes used in these experiments (Figure 4C). By contrast, we observed that both the double *Vav2*^–/–^; *Vav3*^–/–^ and the triple *Vav1*^–/–^; *Vav2*^–/–^; *Vav3*^–/–^ knockout animals exhibit a dramatic reduction in the number of inflammatory cells within the synovial tissue 7 days after the Zymosan A injection (Figure 4B,D). This defect is probably caused by a faster resolution of the inflammatory response, because higher numbers of neutrophils are observed in shorter timepoints upon the Zymosan A administration (Figure 4E). No changes in the numbers of inflammatory cells are observed in the case of *Vav1*^–/–^ mice under the same experimental conditions (Figure 4D).

**Figure 5:**
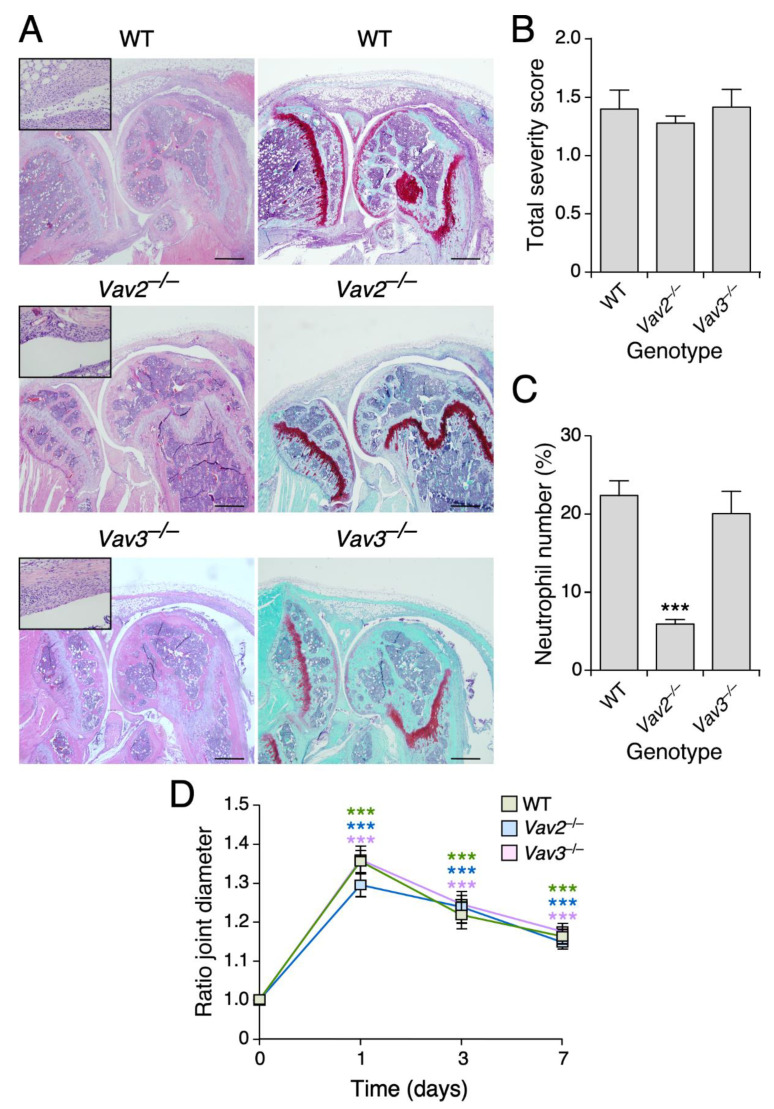
Vav2 is the family member required for sustained inflammatory responses in Zymosan A-treated joints. (**A**) Representative images of histological sections of arthritic knee joints of mice of the indicated genotypes stained with either HE or Safranin-o Fast Green. Scale bar, 500 µm. (**B**) Histological scores for arthritis severity obtained by evaluating hyperplasia, inflammation and cartilage damage on day 7 after intra-articular Zymosan A injection. (**C**) Quantification of the neutrophils infiltrated into the synovium, expressed as the percentage of neutrophils versus synovial fibroblasts. (**D**) Ratio joint diameter results of the measurement of arthritic and control knee joints at the indicated time points. Data shown in panels B to D represent the mean ± SEM. ***, *p* ≤ 0.001 relative to values obtained at time 0 (**D**) or in WT controls (**B**,**C**). *n* = 13 (WT), 12 (*Vav2*^–/–^) and 11 (*Vav3^–/–^*) mice.

To investigate whether this phenotype requires Vav2 and/or Vav3, we performed a new series of experiments using single *Vav2*^–/–^ and *Vav3*^–/–^ mice. We observed that the Vav2 deficiency fully recapitulates the defects previously found in the compound knockout mice (Figure 5A–D). By contrast, the *Vav3*^–/–^ mice behave as WT controls in all these parameters (Figure 5A–D). These results indicate that Vav2 is important for maintaining sustained neutrophil numbers during the development of this type of experimental innate immune-dependent rheumatoid arthritis.

## 4. Discussion

In this study, we have demonstrated that Vav1 and Vav2 play specific roles in different types of rheumatoid arthritis models. In the case of Vav1, we found that it plays quite relevant roles in mBSA-induced arthritis, a monoarticular inflammatory model that recapitulates most of the pathobiological features seen in human rheumatoid arthritis, such as leukocyte infiltration, pannus formation, cartilage destruction and synovial tissue hyperplasia [24]. It is quite likely that the impact of the Vav1 deficiency in this type of arthritis is due to the critical role of this protein during the development and selection of T cells within the thymus. The impairment of these functions causes a mild lymphopenic state in the case of *Vav1*^–/–^ mice [2,17,25,26,27]. However, our data suggest that Vav1 can also contribute to this type of arthritis through the regulation of the proliferation and the differentiation of effector T cells in post-thymic stages (Figure 3). In agreement with this idea, we have seen that the protection against this type of arthritis is not increased upon the genetic depletion of the three Vav family members despite the fact that the lymphopenic state is highly accentuated (25- to 50-fold) in this case [27]. Likewise, we have observed that the activation of transcripts encoding arthritis-associated cytokines (*Tnfa*, *Ifng*, *il1b*) is totally blocked in the joints of mBSA-treated *Vav1*^–/–^ mice (Figure 2B, top panels) although a significant infiltration of *Vav1*^–/–^ CD4^+^ T cells is still observed under such conditions in the joint tissue (Figure 2A). We have also observed that Vav2 is specifically involved in the maintenance of the neutrophil long-term count in the case of Zymosan A-driven arthritis. This protection is very mild, given that the genetic ablation of Vav2 cannot block the swelling and the deterioration of the joint. The same disease evolution is seen in *Vav1*^–/–^; *Vav2*^–/–^; *Vav3*^–/–^ knockout mice, indicating that the mild phenotype found in *Vav2*^–/–^ mice is not due to compensation events by the other two family members. Interestingly, it has been previously reported that the triple Vav family deficiency protects against arthritis caused by the K/BxN serum transfer, an experimental model heavily dependent on neutrophil function [13]. The specific contribution of each Vav family member to this disease was not addressed in that study, so it is unclear currently whether the development of this disease requires Vav2 alone or the rest of Vav family members. In that study, the protection against arthritis was attributed to defects in both the spreading and degranulation of neutrophils lacking the three Vav family proteins. By contrast, and in agreement with our present work, that study did not find any overt defects in neutrophil migration [13]. In line with those previous observations, it is conceivable that the observed decrease in neutrophil infiltration observed in *Vav2*^–/–^ knockout mice could be caused by an impaired adhesion and survival instead of infiltration deficiencies. The different outcome found in these two experimental models is probably due to the type of specific effects elicited by Zymosan A and the K/BxN serum transfer in the joint [23,28,29,30,31]. Given these different outcomes in these experimental models, it is clear that the requirement of the Vav family function in this disease will be highly dependent on the experimental conditions used to trigger the arthritic condition.

To our knowledge, this is the first study demonstrating that the elimination of a single Vav family member confers full protection against antigen-induced arthritis. This might be relevant in the context of human disease, since recent studies have revealed a possible link between a specific *VAV1* SNP (rs2546133) and rheumatoid arthritis severity [10,11]. rs2546133^+^ patients also exhibit extra-articular manifestations such as amyloidosis, Sjogren syndrome and vasculitis [10]. Likewise, there is a correlation between four *VAV1* SNPs (rs682626-rs2546133-rs2617822-rs12979659) and the development of a rheumatoid arthritis condition negative for antibodies to citrullinated circular peptides [11]. Some of those SNPs (rs2546133-rs2617822) are also associated with multiple sclerosis [12], a disease that shows significant overlaps in terms of genetic predisposition and molecular features with rheumatoid arthritis [32]. It has been shown that this risk SNP is associated with high levels of VAV1 protein in patients. It is also associated with increased expression of both TNFα and IFNγ in the peripheral blood and cerebrospinal fluid of the patient samples [12,33]. Interestingly, the expression of these two cytokines, which is associated with the development of the arthritic condition as well [34], is also severely impaired in the case of the mBSA-injected joints of *Vav1*^–/–^ mice (Figure 2B).

Taken together, the data presented in this work suggest that the use of inhibitors for the catalytic activity of Vav1 could represent a viable alternative to reduce the degenerative processes associated with rheumatoid arthritis and related diseases, such as multiple sclerosis, at least in their most incipient states. Other inhibitory strategies can also be explored, including the use of peptide and RNA aptamers as well as proteolysis targeting chimera-based methods. Although Vav1 plays both catalysis-dependent and independent functions [1,2], current genetic evidence suggests that the elimination of the catalytic activity is self-sufficient to block most of the physiological functions of Vav1 in T cells [35]. It is clear, however, that the use of therapeutic strategies against the whole protein would allow the complete elimination of all the spectrum of Vav1 downstream signaling branches. More work on Vav1 will be required to address these issues.

## Data Availability

The data underlying this article are shared on reasonable request to the corresponding author.
